# FRAMINGHAM HEART STUDY NOVEL EXAMINATION USING TECHNOLOGY IN COMMUNITY-DWELLING ADULTS

**DOI:** 10.1093/geroni/igz038.1356

**Published:** 2019-11-08

**Authors:** Joanne Murabito, Joanne M Murabito, Ludovic Trinquart, Emelia J Benjamin, Honghuang Lin, Nicole Spartano, Mayank Sardana, David D McManus

**Affiliations:** 1 Boston University, Boston, Massachusetts, United States; 2 Boston University School of Medicine, Boston, Massachusetts, United States; 3 Boston University School of Public Health, Boston, Massachusetts, United States; 4 Boston University School of Medicine, Framingham, Massachusetts, United States; 5 Boston University, Framingham, Massachusetts, United States; 6 University of Calfornia San Francisco, San Francisco, California, United States; 7 University of Massachusetts Medical School, Worcester, Massachusetts, United States

## Abstract

The electronic Framingham Heart Study (eFHS) is a mobile health cardiovascular monitoring system deployed at the current in-person exam. We developed a smartphone app which includes an electronic consent, health questionnaires, and integrates with a wireless blood pressure (BP) cuff and Apple Watch. We have deployed the app in 1927 FHS participants (mean age 52.8, range 32-80 years, 57% women, 7% multi-ethnic) who owned a compatible smartphone (iPhone or Android). We have observed high rates of initial survey adherence (85% completed baseline surveys). Preliminary results demonstrate about 76% of participants who transmit data from the Apple Watch continued to transmit data 12 weeks following enrollment. Our experience highlights the capacity of FHS participants to transmit meaningful mHealth data. We plan to identify factors associated with long-term use and to compare digital data to data collected in the Research Center to understand the value of this novel means of data collection.

